# Supplementary material for: Autoclave reactor synthesis of upconversion nanoparticles, unreported variables, and safety considerations
Source: Commun Chem. 2025 Feb 6;8:36. doi: 10.1038/s42004-025-01415-3 (PMC11802760; doi:10.1038/s42004-025-01415-3)
Supplement: Supplementary file 2 — Supplementary Information [file 42004_2025_1415_MOESM2_ESM.pdf]

SUPPLEMENTARY INFORMATION

Autoclave reactor synthesis of upconversion nanoparticles, unreported variables, and safety considerations

Rebecca McGonigle,[†] Jodie Glasgow,[†§] Catriona Houston,[†] Iain Cameron,[†§] Christian Homann,[††] Dominic J. Black,[‡] Robert Pal,[‡] Lewis E. MacKenzie[†]*

[†]Department of Pure and Applied Chemistry, University of Strathclyde, Glasgow, G1 1RD, United Kingdom.
[§] Now at the Department of Chemistry, University of Manchester, Manchester, M13 9PL, United Kingdom.
[††] Division of Biophotonics, Federal Institute for Materials Research and Testing (BAM), D-12489 Berlin
[‡] Department of Chemistry, Durham University, Durham, DH1 3LE, United Kingdom.

*Corresponding author. Email: l.mackenzie@strath.ac.uk

Section A. Historical trends in UCNP research

From the 1960s, photonic upconversion research mainly focused on bulk upconversion materials, with $NaYF_4$:Yb,Er emerging as the upconversion material of choice.[1] In the mid-2000s, UCNPs started appearing in the literature, correlated with, albeit lagging behind the more general term *"nanoparticles"* (see Figure S1). Up until 2017, the number of publications featuring "*upconversion nanoparticles*" increased exponentially year-on-year, tracking the wider report of publications featuring "*nanoparticles*". This pattern is mirrored for related search terms such as *"UCNPs"* and *"upconversion nanocrystals"* (data not shown here). Subsequently, the number of publications on UCNPs appears to be declining year-on-year by a modest amount. This stands in contrast to the wider field of "*nanoparticles*", which appears to be reaching a plateau, perhaps indicating the maturity of the field of UCNPs after an initial exponential rise.

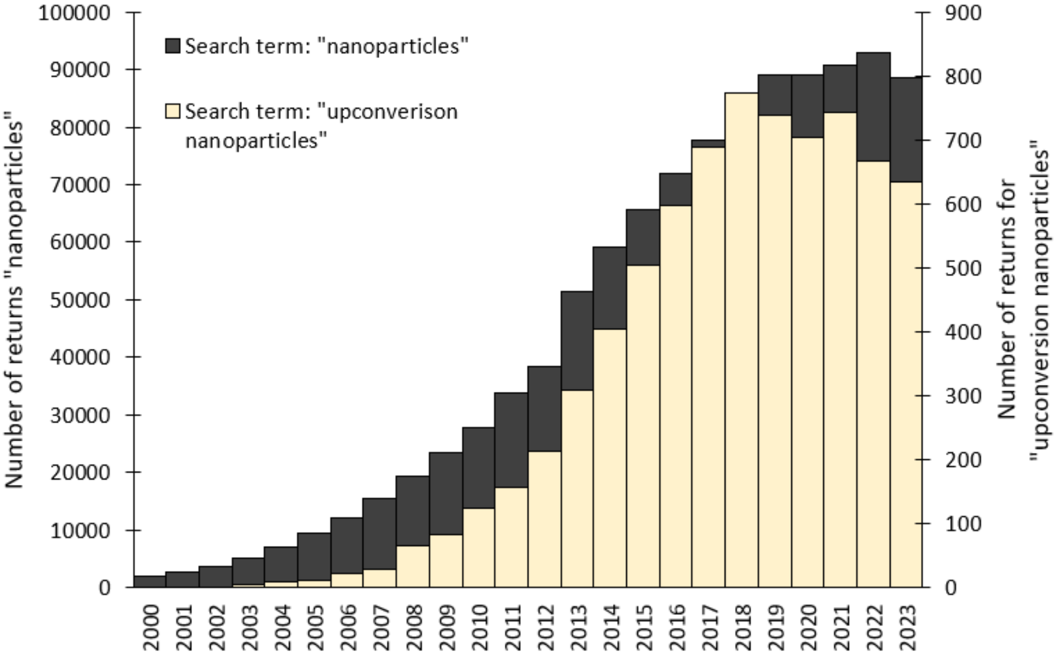

Figure S1. The annual number of publications indexed by *Web of Science*™ featuring the term *"upconversion nanoparticles"* (yellow bars) vs the number of publications featuring the search term *"nanoparticles"* (black bar). Data Copyright Clarivate 2024, all rights reserved. Figure is original.

Section B. Expense of purchasing UCNPs

Despite their utility, UCNPs are extremely expensive to purchase. For example, in mid-2023, a leading chemical supplies company listed 10 mg of OA coated $NaYF_4$:Yb,Er UCNPs at a cost ~£320 + 20% VAT. From the same major supplier, core/shell $NaYF_4$,Yb,Tm@$NaYF_4$,Yb,Nd UCNPs with dual-wavelength excitation (808 nm and 976 nm) were listed at £780 + VAT for 10 mg. Further, core/dual-shell UCNPs with a $NaYF_4$:Tm core, a passive $NaYF_4$ shell, and a silica outer shell will cost £5940 + VAT for 10 mg. These costs will likely make purchasing the required quantities of UCNPs for various applications prohibitively expensive. Further, despite the price of UCNPs, it seems that major chemical supply companies offer little in the way of quality assurance or continuous availability of UCNPs. For instance, the aforementioned leading chemical supplies company only stated UCNP surface ligands, approximate diameter, and peak of emission waveband(s). There is no information about UCNP size distribution, no representative UCNP spectrum, and no electron microscopy images demonstrating UCNP morphology: all very important parameters for UCNP applications. Further, during the process of writing this manuscript, the majority of the UCNPs products discussed herein were withdrawn from the market without notice to consumers. This highlights the unpredictability of sourcing UCNPs commercially.

When considering commercial procurement of UCNPs, it is necessary to consider what quantity is needed. For example, quantum yield estimation typically requires 75 mg of UCNPs.[2] Whereas for *in vivo* studies, doses of 100 mg/kg of bodyweight may be administered to a mouse. Given that a typical mouse may weigh ~20 g, this requires only 2.5 mg of UCNPs. However, such studies may require repeated UCNP dosage for many days with many animals, the quantity of UCNPs required will quickly add up. A good example is an *in vivo* biocompatibility/UCNP tolerance study reported by Zhou et al., (2019)[3] in which $NaYF_4$:Yb/Er@SiO_2 UCNPs were administered to 36 mice (average weight 22 g) for up to 14 consecutive days at UCNP doses of 20 mg/kg and 100 mg/kg of bodyweight. We estimate that this study would have required ~360 mg of $NaYF_4$:Yb/Er@SiO_2 UCNPs, assuming zero wastage. Assuming $NaYF_4$:Yb/Er@SiO_2 UCNPs could be procured at a cost of £700 for 10 mg (excluding V.A.T.) we estimate that 360 mg of such UCNPs would cost in excess of £25,000. This is an extremely large cost for research consumables.

Ultimately, these factors combine to make commercial procurement of UCNPs extremely risky and expensive for a research project. Consequently, reliable and repeatable on-demand synthesis of UCNPs is not only desirable, but absolutely necessary for reproducible UCNP studies across the sciences.

Section C. Low-temperature UCNP synthesis techniques

This perspective is focused on synthesis methods that produce luminescent UCNPs in colloidal suspensions. Therefore, we have excluded bulk and microscale upconversion materials, including any process involving high-temperature annealing.[4] Ball milling and continuous reactor synthesis of UCNPs are covered by Jiao et al., (2020).[5] Additionally, there are some intriguing room-temperature UCNP synthesis techniques which are under-explored and do not strictly fit the scope for the wider context of this perspective. Therefore, these are highlighted as supplementary information.

Ultrafast (~1 minute) room-temperature synthesis of bare $NaBiF_4$:Ln upconversion nanomaterials was reported by Lei et al (2017)[6] and Du et al., (2018).[7] In our own (unpublished) attempts to reproduce these methods, we found that upconversion emission from such materials in EtOH was detectable, but rather weak, possibly due to solvent quenching. One could envision scenarios where upconversion of such UCNPs could be enhanced by (a) simple dispersal in non-polar solvents, or (b) forming a protective inert shell or polymer layer around the $NaBiF_4$:Ln UCNPs. Nevertheless, room temperature synthesis of UCNPs is exceptional as UCNP synthesis typically requires temperatures between 180 and 310 °C.[5]

Shao et al., (2014)[4] reported a layer-by-layer nanosheet process for forming beta-phase (hexagonal) $NaYF_4$:Ln UCNP nanorods with a 3 hour reaction step at the exceptionally low temperature of 50°C.[4] These $NaBiF_4$:Ln

UCNPs reportedly showed upconversion emission in EtOH. Intriguingly, Liang et al., (2007)[8] reported formation of α-phase $NaYF_4$ nanocrystals at room temperature, with a very indistinct X-ray diffraction pattern, indicating poor crystallinity; upconversion emission via this room-temperature host lattice formation was not investigated.[8] More recently, Lei and Zhang (2021)[9] produced hollow $NaBiF_4$:Yb,Er UCNPs in a one-pot room-temperature synthesis via a "liquid nanoparticle" approach, although it is unclear if these UCNPs are suitable for dispersal in solvents.[9] Further investigation of these synthesis methods could be beneficial for "green" UCNP synthesis or synthesis of UCNP at larger scale.

Section D. PVP-assisted UCNP synthesis

Perhaps the simplest route of producing UCNPs is the polyvinylpyrrolidone (PVP) assisted route, which requires only basic laboratory equipment to facilitate the reaction involved. The PVP UCNP synthesis route was first reported by Li and Zhang in 2006,[10] who proposed a UCNP formation mechanism where Ln^{3+} ions coordinate with the hydrophilic pyrrolidone moiety of PVP, thus forming nucleation sites for UCNP growth. The primary reaction in this procedure can be conducted open-to-air in round bottom flasks (160 °C for 2 hours) with the simple hot-plate stirring/heating apparatus that is readily accessible in many wet laboratories. We have found this method typically produces ~130 mg of PVP-coated UCNPs with a typical diameter of ~50 nm.[10–12] These PVP UCNPs can be dispersed in a wide range of solvents, e.g. water, ethanol, methanol, isopropanol, chloroform, DMSO, DMF, etc; and can also be functionalised with silica shells.[10] A wide range of emission colours have been demonstrated, including red, green, and blue emission achieved via co-doping with various combinations of Yb^{3+}, Er^{3+}, Tm^{3+}, and Mn^{2+}.[10–12] Typically PVP-UCNPs appear pseudo-spherical or pseudo-cuboidal, but their morphology can be altered by Mn^{2+} co-doping, producing smaller and less homogenous UCNPs that may indicate a non-uniform UCNP formation process, similar to hollow UCNPs reported by others.[9] Scale-up of PVP-UCNP synthesis could likely be achieved via parallel batch synthesis or a with a larger reaction vessel; attractive for large-scale applications such as light-harvesting in solar cells, display technologies, commercial biosensors, and security inks.[12,13]

Jin et al., (2011)[14] noted that PVP-coated UCNPs had good biocompatibility.[14] However, the PVP-UCNP synthesis does have some inefficiencies that should be noted. Firstly, it involves a seemingly redundant heating and drying step to convert lanthanide oxides (Ln_3O_2) in 10% HNO_3 to lanthanide nitrates ($Ln(NO3)_3$). This is inefficient because the lanthanide oxides are poorly soluble in 10% HNO_3 and so it may take some time (on the order of days) for full dissolution of concentrated stock solutions. It is apparent that starting with readily soluble lanthanide nitrates would be more efficient. However, in our experience this does not result in the formation of PVP-UCNPs for unclear reasons. Secondly, the degradation of nitrates under heating produces orange-coloured fumes, likely the pollutant NO_2.[12,15] Thirdly, whilst convenient, the open-to-air synthesis may result in oxygen vacancies in the $NaYF_4$ host lattice (i.e. oxygen competing with fluorine) thereby altering/reducing upconversion efficiency.[16] Finally, the origin of rhombus-shaped PVP-UCNPs observed by Birch et al., (2023)[11] is unclear. Electron microscopy diffraction and non-quantitative elemental mapping analysis indicated that these rhombus-shaped nanoparticles are composed of cubic-phase $NaYF_4$:Yb,Er. However, rhombus shaped nanoparticles are more commonly associated with YF_3 structures that are formed in reactions where Na^+ is scarce. Further research into the formation of the rhombus-shaped PVP-UCNPs is warranted, and may benefit from single-nanoparticle analytical techniques.[11,17] In the broader context, the quantum yield of PVP-UCNPs has not yet been measured and benchmarked against other upconversion materials; action is needed to address this important point of comparison.

Section E. Microwave synthesis of UCNPs

Microwave synthesis of UCNPs is less well established than autoclave synthesis or co-precipitation methods, yet it is highly attractive because contemporary microwave reactors offer straight-forward operation, excellent control of synthesis variables, and short reaction times (e.g. 1 second to 10 minutes for enclosed reactors with pressurised reaction vessels).[18] In microwave UCNP synthesis, the reaction mixture is contained in reinforced closed reaction vessels capable of withstanding elevated temperatures and pressures to similar tolerances of autoclaves. For example, some MARS 6 (CEM Corporation) reaction vessels can reportedly handle pressures of up to 55 bar at 240 °C or 7 bar at 300 °C.[19] Additionally, multiple reaction vessels are often housed in a rotating carousel, or handled via a robotic arm autosampler, allowing parallel or sequential batch production of UCNPs in a convenient manner. Heating is induced via microwave dielectric heating of the reaction mixture – which requires careful consideration, especially where there is little to no water in reactions.[20] Contemporary microwave reactors will offer in-line temperature monitoring and data reporting, which is highly beneficial for reproducibility - see the recently reported example by Egatz-Gomez et al.[21] Some microwave reactors may even enable visual monitoring of the reaction mixture via camera systems. Ultimately the potential for batch production of UCNPs in minutes at the push of a button, makes microwave UCNP synthesis highly desirable.

The current downsides to microwave synthesis of UCNPs are that: (a) laboratory-grade microwave reactors require considerable capital investment (see Table 1), (b) microwave synthesis is somewhat less well established in terms of the body of the literature (meaning that not all host lattices are available), and (c) reaction scale is limited by the size of reaction vessels (typically 20 or 35 mL). There are some unique variables to consider in microwave synthesis of UCNPs. For example, choice of solvent plays a crucial role in how products are heated. Polar solvents (e.g. water, ethylene glycol, benzyl alcohol) are heated efficiently via microwaves due to these solvents offering good microwave absorption; however, use of these solvents generally produce larger nanoparticles. Examples of solvents used in microwave UCNP synthesis include aqueous solutions,[22,23] mixtures of OA, ODE, olamine (OAm)[24]; and bis(2-ethylhexyl) adipate (BEHA).[21] How efficient a solvent is regarding microwave absorption and heating is an important aspects, with OA and ODE being poor absorbers of microwaves.[20] As an aside, gum Arabic has recently been used to render OA-capped microwave synthesised UCNPs water dispersible via emulsification; a convenient procedure for printing UCNP security inks using commercial jet printers.[25]

Notably, UCNP precursor selection may also vary the heating rate achieved.[20] It is also known that different precursors (e.g. [Ln(TFA)$_3$], [Ln(OA)$_3$], [Ln(Ac)$_3$]), thermally decompose at different temperatures, resulting in nucleation of UCNPs at different temperatures, altered pressure during the course of UCNP synthesis, and ultimately differences in final UCNP size.[24] Some examples of UCNPs produced by microwave synthesis include: sub 5 nm Na(Gd-Yb)F_4:Tm UCNPs [20]; sub-10 nm core/shell β-phase $NaGdF_4$:Yb,Er UCNPs (reaction time between 1 second to 10 minutes)[24]; microparticles with upconversion and downshifting capabilities ($LiYbF_4$, $NaYF_4$, $NaGdF_4$, $LiYF_4$ host lattices with various emissive dopants, i.e. Yb^{3+},Er^{3+} – Yb^{3+},Tm^{3+} – Ce^{3+},Tm^{3+})[22]; $NaYF_4$:Yb/Er with subsequent mesoporous silica coating[23]; LaOF:Yb^{3+},Ho^{3+} UCNP nanorods[26]; and rhombus-shaped GdF_3:Yb,Er UCNPs.[27] Aside from UCNP synthesis, microwave reactors are often used for digestion of both organic and inorganic samples via acid treatment.[28,29] Reputable manufacturers of microwave reactors include Anton Parr GmBH (Germany) and CEM Corporation (NC, USA).

Overall, microwave synthesis of UCNPs produces high-quality UCNPs in a highly controlled manner on a short timescale, with ample opportunity for parallel synthesis in multiple reaction vessels. Microwave reactions require careful experimental design to achieve desired synthesis results, which can't be readily translated from other synthesis methods, however, there is an expanding body of literature on suitable methods. Like autoclave synthesis, key variables such as reactor and reaction chambers used should be reported for microwave

synthesis techniques to be reproducible. Ultimately, microwave synthesis is a highly attractive UCNP synthesis route.

Section F. Hot-injection/Thermal co-precipitation synthesis of UCNPs

Hot-injection synthesis (also known as "thermal decomposition" or "thermal co-precipitation") offers the most control over form and quality of UCNPs produced albeit at the requirement of a high-degree of operator skill. For example, it can be used to make extraordinarily sophisticated $NaErF_4$:Tm core/multi-shell UCNPs with various six-shell layers,[30] small and bright UV-emissive $LiYbF_4$:Tm@$LiYF_4$ core/multi-shell UCNPs,[31] and $NaYF_4$:Yb,Er, @$NaYF_4$ core/shell UCNPs with record-breaking quantum yields.[32]

The hot-injection method is demanding. It requires multiple three-necked round bottom flasks, a gas control system (e.g. a Schlenk line with appropriate vacuum pumps and liquid nitrogen cooling traps), one or more syringe pumps, a high-temperature heating mantle, and a skilled operator experienced enough to safely manipulate these components. As a general example of hot-injection synthesis of the most common upconversion material, i.e. $NaYF_4$ based, rare-earth salts are dissolved in a mixture of water and acid to form precursors. These precursors are then introduced into OA in a separate reaction flask under vacuum or inert atmosphere, which is then heated at elevated temperature (e.g. 100 – 120 °C) to form rare-earth oleates. In the next step, a cation source such as Na^+ (or alternatively Li^+, Ca^{2+}, Ba^{2+}),[33,34] is added to the oleates, and after another cycle of degassing and dissolving, the hot solution is transferred to a heated syringe and injected into a pre-degassed mix high boiling-point solvents at elevated temperature (e.g. OA/ODE/OAm at 300 – 360°C). This reaction mixture is kept at the elevated temperature for several hours, with precursors decomposing to form the core UCNPs. The core UCNPs are then collected via centrifugation and washed. Subsequent layers of shells are formed around UCNPs by repeating similar reactions with UCNPs present and injecting new precursor materials. [31,35]

There are a number of variables to consider in hot-injection synthesis. For example, OAm can be incorporated as a less strongly coordinating solvent, and ODE can be used as a non-coordinating solvent; altering the ratio of OA, OAm, and ODE will alter the morphology of UCNPs produced.[31] Likewise, the precursor, their ratios, addition rates, and water content can all affect crystal structure, morphology, and properties of the resultant UCNPs.[36] Production of the highest-performance UCNPs requires diligent choice of precursors, dry (i.e. water-free) solvents, and a sophisticated approach wherein precursor cubic-phase UCNPs are redissolved in fresh solvent to obtain hexagonal-phase UCNPs.[32] Given that the reaction occurs in a single reaction vessel, hot-injection UCNP synthesis is inherently inefficient for exploring the parameter space of UCNP composition.

Andresen et al., (2023)[37] have produced a detailed study of the reproducibility of UCNPs produced by the hot-injection method at various scales. They found that despite the complexity of mass transport dynamics and seed nanocrystal formation, the hot-injection reaction could be upscaled to 5 g per batch. You et al., (2018)[38] also demonstrated a hot-injection synthesis of beta-phase core/shell UCNPs at over 60 g per batch.[38] Therefore, despite its complexities, hot-injection UCNP synthesis is attractive for large-batch production of UCNPs.

Ultimately, the hot-injection UCNP synthesis method may be viewed as the highest-performance UCNP synthesis technique because it enables production of exceptional quality core/shell UCNP products. However, it requires highly skilled personnel with a strong chemistry background to control all the variables involved and to undertake air and water-free reactions safely and, more fundamentally, only a single reaction can be undertaken at a time with typical apparatus.

Section G. Automated UCNP synthesis, machine-learning, and self-driving fluidic labs
It is worth noting that if financial resources are not a limiting factor, fully-automated approaches to UCNP synthesis have been demonstrated.[39–43] These approaches require custom-designed robots with the ability to weigh compounds, mix liquids, and heat many vials at once up to 325 °C in oxygen-free environments.[44] Such automated systems massively reduce time to results where many variables are involved. For example, the screening of 78 UCNP dopants combinations (as demonstrated in Chan et al., (2012)[39]) would require around 4 months of continuous synthesis work with a standard single-chamber autoclave reactor (assuming one synthesis cycle per day due to heat-up/cool-down times), but would likely take less than a single day to achieve with an automated nanomaterial synthesis system. This time advantage of automated nanomaterial synthesis laboratories enables exploration of sophisticated nanoarchitectures.

At the time of writing in 2024, we are experiencing a nascent epoch of machine learning approaches being applied to scientific challenges, with nanoparticle engineering being no exception. Recently, Xia et al., (2023)[45] reported machine learning-driven approaches have recently been reported to theoretically optimise multi-shell UCNP composition and architecture via simulation.[45] On the practical side of nanomaterial synthesis, Bateni et al., (2024)[46] reported that a closed-loop "self driving fluidic lab" produced record breaking Mn-Yb co-doped $CsPbCl_3$ quantum dots with a photoluminescence quantum yield of 158%.[46] Such approaches have huge advantages not only in quality of output products, but also in terms of material cost and labour time. Given that the self-driving fluidic lab incorporates atmospheric control, pressure containment, and high-temperature synthesis, it seems likely that it will only be a matter of time before such self-driving fluidic lab approaches are turned to the production of UCNPs.

Section H. Case study #1: adapting a hybrid hydrothermal/solvothermal autoclave synthesis method to produce oleic-acid coated UCNPs (OA-UCNPs)
There is abundant literature on the hydrothermal synthesis of oleic-acid functionalised UCNPs (OA-UCNPs) using autoclaves. However, there are two major problems with reproducing hydrothermal OA-UCNP synthesis: (1) the underlying principles of the hydrothermal synthesis are usually not described, and (2) many autoclave synthesis variables – as outlined in Table 2 – have been historically unreported. In our experience, this has made it challenging and tedious to reproduce autoclave hydrothermal syntheses reported by other groups. Rather, published approaches have served as a starting point, with a great deal of refinement and optimisation research required to produce viable OA-UCNPs. In this case study we aim to convey an example of this process, the frustrations encountered, and the "lessons learned".

For the purposes of this case study, we adapted a procedure from Bi et al., (2022),[47] which reports the synthesis of cubic shaped $NaYF_4$:Yb,Er,Mn UCNPs with strong single-band red emission via hydrothermal autoclave reaction. The choice to adapt Bi et al., was somewhat arbitrary as we were simply aiming for monodisperse UCNPs ~50 nm in diameter with strong red emission. Importantly, in this scheme precursors are lanthanide chlorides ($LnCl_3$), and the Mn source is $Mn(CH_3COO)_2$. The reaction includes a small amount of aqueous 2M NaOH (1.5 mL), 10 mL "anhydrous alcohol", and 5 mL OA. It is believed that these reactants combine to form Na and RE oleates.[48] Rather than being purely hydrothermal, this is a biphasic system consisting of small reverse micelles of water within OA (and ODE if present)(see Figure S2).[48,49] Understanding the biphasic nature of "hydrothermal" UCNP synthesis was key to our understanding and control of the UCNP synthesis reaction, which we herein refer to as a "hybrid hydrothermal/solvothermal" reaction. At a suitably high temperature, the $Mn(CH_3COO)_2$ precursor thermally decomposes, resulting in UCNP nucleation events (N.B. precursor choice is an important topic in UCNP synthesis and is best covered in-depth by others).[24,50]

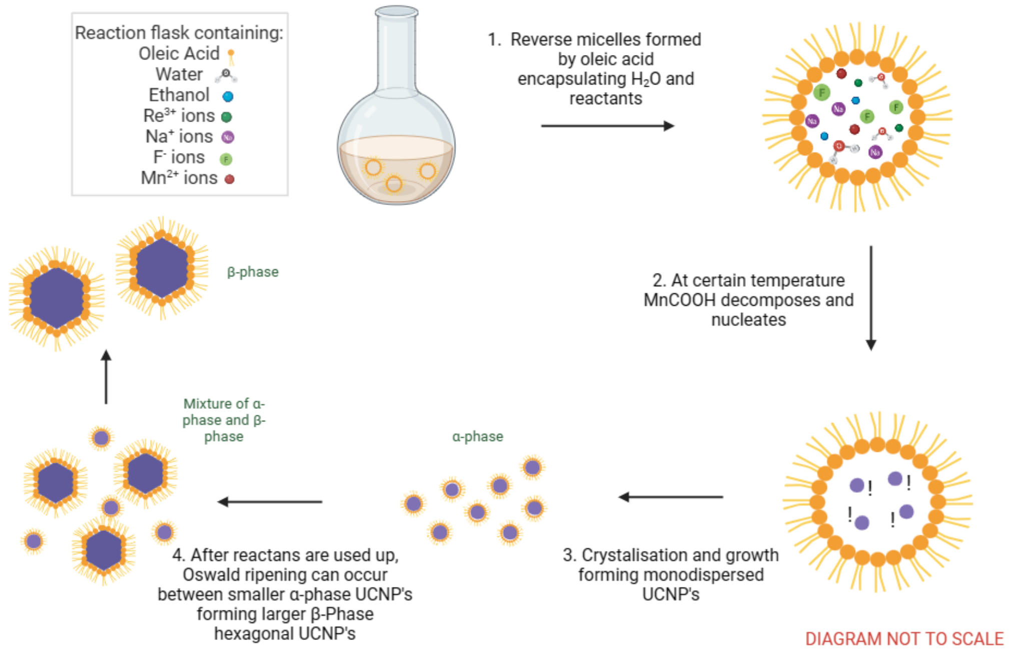

Figure S2. Hybrid hydrothermal/solvothermal reaction scheme for producing OA-UCNPs.

Like most studies in the field, Bi et al., gave a sparse description of their autoclave methods: i.e. "*a 50 mL stainless Teflon-lined autoclave*" which was "*continuously heated at 200°C for 8 hours*". We set about to reproduce results from Bi et al., for $NaYF_4$:Yb,Er,Mn (25 mol% Mn^{2+}) UCNPs using our Asynt PressureSyn high-safety autoclave reactor (see Figure 1b). In our initial procedure, we prepared stocks of NaOH, $RECl_3.6H_2O$, $Mn(CH_3CO_2)_2.4H_2O$, and NaF in deionised water and appropriate amounts were mixed with EtOH (Bi et al., specified "anhydrous alcohol"),[8] and OA; these were then stirred thoroughly before addition to the autoclave. Our use of stock solutions resulted in the addition of 10 mL more water than in Bi et al., where solid $RECl_3$ and $Mn(CH_3CO_2)_2$ starting materials were added directly to the reaction solution. Our Pressure Syn autoclave reactor system was a stainless steel autoclave reactor with a 125 mL total chamber volume and a lidless liner with a total maximum volume of ~80 mL volume. Heating was provided by a hotplate (IKA RCT) and a specialised heating mantle. The manufacturer states that this system provides uniform heating to the bottom and sides of the autoclave reactor. An internal thermocouple attached to the autoclave was immersed within the reaction solution and was used to provide temperature control and feedback. All starting materials and solvent volumes were kept as close as possible to Bi et. al., and an atmospheric blanket of N_2 was introduced before sealing the autoclave reactor. Prior testing had indicated that reaction mixtures that are predominately OA would reach a maximum of 180°C in our autoclave reactor. Therefore, we initially set the target temperature to 190°C to maximise heating rate (or to 200 °C if necessary), subsequently lowering the target temperature to 180°C once the reaction mixture reached 180°C. The reaction timer was started once the autoclave reactor internal temperature reached 120°C (after ~30 minutes of heating) and the reaction subsequently proceeded for 8 hours before heating was disabled and the reactor was allowed to cool naturally. The reaction mixture was not stirred. Pressure and temperature data was logged manually. The variation of reactor temperature, reactor pressure, heating mantle temperature, and hotplate output temperature are shown in Figure S3. After the reaction was complete, the products were recovered by centrifugation, washed, and analysed with electron microscopy and upconversion spectroscopy. The resulting UCNPs were curious: they exhibited strong red upconversion, but the nanoparticles were a mixture of nanobeans and flat plate shapes (see Figure S4). This was entirely unlike what was expected from Bi et al., (2022) and coincidentally more like LaF_3:Yb,Er nanoplates reported Liu et al.,

(2007).[51] Therefore, the reaction conditions required careful consideration and modification to achieve a well controlled synthesis.

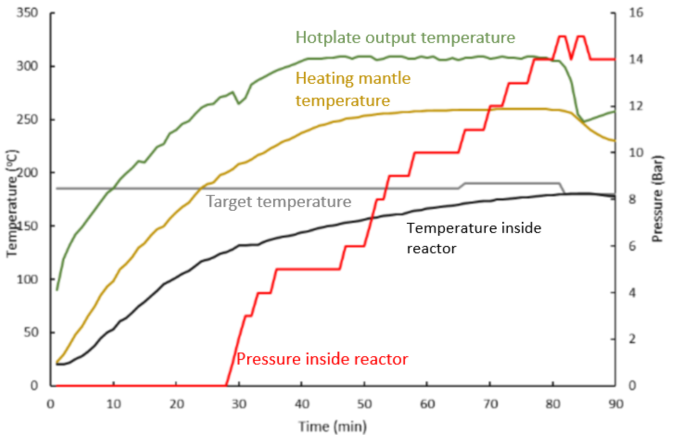

Figure S3. Heat and pressure of our Asynt PressureSyn autoclave reactor system during the initial phase of a typical hybrid hydrothermal/solvothermal UCNP synthesis.

Due to the biphasic nature of the hybrid hydrothermal/solvothermal reaction, we hypothesised that the reaction could be improved by (a) removing as much water as possible from the synthesis and (b) purposefully and sequentially adding starting materials. First, 1.5 mL NaOH was mixed with OA for 1 hour by stirring at 700 RPM to form sodium oleates. Then stocks of $RECl_3.6H_2O$ pre-prepared in EtOH were added to the mixture. Finally, solid $Mn(CH_3CO_2)_2.4H_2O$ and NaF were added directly and slowly, before further stirring. We propose that this process mainly confines the majority of UCNP precursor material – including the important $Mn(CH_3CO_2)_2$ for thermal decomposition/nucleation – to within the reverse micelles. However, the reduced water content also reduced total reaction mixture volume which was problematic because the thermocouple probe (introduced via an angled port) could not be immersed into the reaction mixture! Therefore, the heat applied to the autoclave was controlled via the temperature of the heating mantle. From prior experience of monitoring both heating mantle and internal temperatures, we surmised that a heating mantle temperature of 200°C would be appropriate for reaching 180°C inside the autoclave reactor. The reaction timer was started once the heating mantle reached 145°C and the reaction was allowed to occur for a variety of time points.[i]

With this updated protocol, instead of the expected UCNP nanocubes, we produced UCNPs in a variety of shapes, dependent on reaction time. For a 2 hour reaction, small irregular "nanobean" shaped UCNPs were produced with a diameter of 26 ± 6 nm (maximum ferret diameter ± standard deviation) (see Figure S4a); 4 hours produced the aforementioned "nanobeans" with the addition of thin hexagonal plates forming (see Figure S4b); 8 hours produced rods with a hexagonal-face and ends that could be described as "crown like" were produced (266 ± 12 nm across the hexagonal face) (see Figure S4c,d). This UCNP formation processes has been reported in a number of other studies: the "nanobeans" are meta-stable α-phase (i.e. cubic) crystal lattice nanoparticles formed at relatively low temperatures, and which re-dissolve at higher sustained temperatures, resulting in the formation of more stable and larger β-phase UCNPs via Ostwald ripening (see references for detailed discussion).[49,50,52–54] When dispersed in cyclohexane, upconversion emission from the larger OA-UCNPs was

[i] An alternative solution to this temperature probe issue would have been to purchase another thermocouple, attach a ferrule higher up, and simply bend the thermocouple as appropriate to reach the bottom of the reaction chamber).

much stronger than the smaller “nanobean” OA-UCNPs; this was attributed to reduction of size-dependent quenching via interaction with solvent molecules.[55,56]

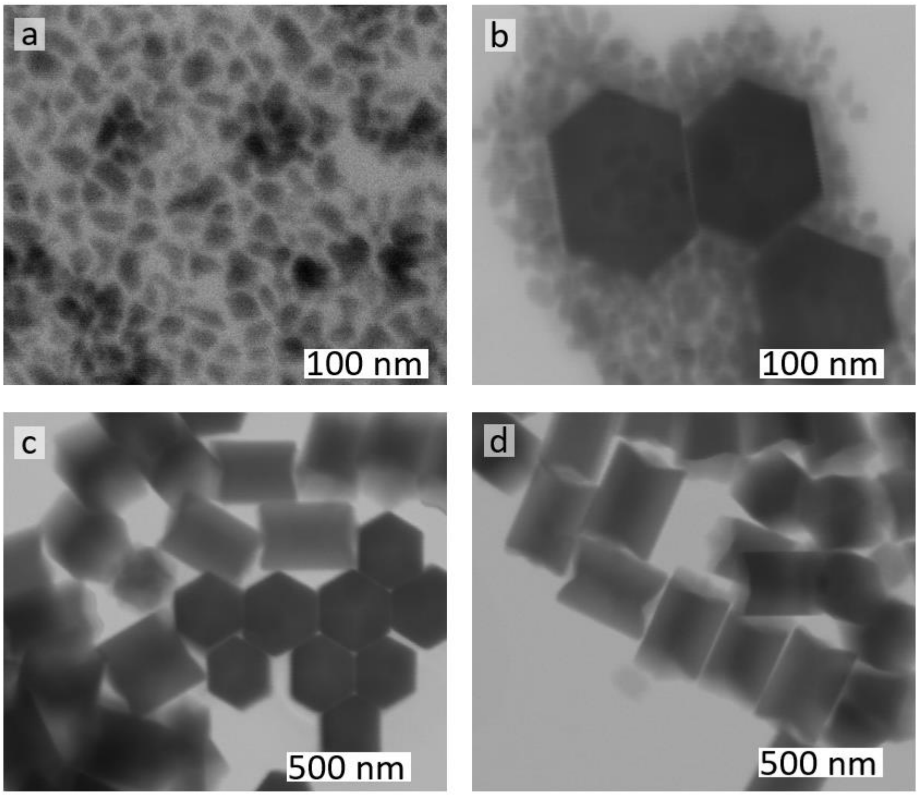

Figure S4. UCNPs produced by the hybrid hydrothermal/solvothermal synthesis described in Case Study #1. (a) 2 hour synthesis product: “nanobean” shaped. (b) 4 hour synthesis product. (c, d) 8 hour synthesis product.

Ultimately, despite successfully producing UCNPs, we spent a large number of person-hours optimising a synthesis procedure that have been well established in the literature since ~2007 onwards. With hindsight, the example set by Liang et al., (2007)[8] indicates that varying the alcohol used in this hybrid solvothermal synthesis from ethanol to methanol may have produced the cube-shaped UCNPs produced by Bi et al., (2022).[47] We also did not explore many variables, such as precursor choice, ratio of various reactants, pH, and ratio of solvents such as OA and ODE. With such a large parameter space to explore, parallel batch synthesis of UCNPs (e.g. via multi-chamber autoclaves or simultaneously heating many autoclaves at once via oven or heat-block) would have been highly advantageous in order to increase research efficiency.

Section I. Case study #2: solvothermal autoclave synthesis of water-dispersible PEI-UCNPs
As a contrasting case study, we present a solvothermal autoclave synthesis method for producing PEI-coated UCNPs. We adapted this solvothermal method from both Zhang et al., (2014)[57] and Nampi et al., (2018)[58] and have found it to be particularly reliable and repeatable. In brief, rare earth nitrate hydrates, NaCl, and PEI polymer (branched PEI, average molecular weight 25,000) are all dissolved and mixed in ethylene glycol. This is combined with NH_4F in ethylene glycol and then transferred to an autoclave. In this procedure, the only sources of water are incidental: arising from the rare earth nitrate hydrate starting materials and the ambient atmospheric humidity (either via direct exposure and/or due to the hygroscopic nature of ethylene glycol and various other starting materials). Further, ethylene glycol is miscible with water, therefore even if water is present, no micelles are expected to be formed and this may be considered a purely solvothermal autoclave

synthesis. Zhang et al., do not detail the autoclave used, whereas Nampi et al., state they used a Parr Pressure Instrument Company autoclave (albeit an unspecified model) with a volume of 120 mL and PTFE liner. Both Zhang et al., and Nampi et al., state that the autoclave is heated at 200°C for 2 hours, but do not state the style of autoclave or how it was heated. We have found that following published protocols in our Asynt PressureSyn autoclave reactor reliably produces PEI-UCNPs with properties (i.e. size, crystal structure, and luminescence) that closely match prior studies (see Figure S5a). For example, our powder x-ray diffraction studies have measured an alpha-phase crystal lattice parameter of 5.47 ± 0.01 Å, which is consistent with the value of 5.47 ± 0.02 Å reported by Nampi et al. Notably, the UCNPs are fairly small (~16 ± 3 nm) with a large hydrodynamic diameter 148 ± 65 nm (measured by dynamic light scattering) and strong positive zeta potential (typically +28 mV) indicating that the PEI polymer enables the PEI-UCNPs to be well dispersed in water. Furthermore, the $NaYF_4$:Yb,Er UCNPs (Yb = 18 mol%, Er = 2 mol %) produced exhibit remarkable unusually strong red emission for $NaYF_4$:Yb,Er UCNPs without Mn^{2+} co-doping; this may indicate that the PEI is protecting the $^4F_{9/2} \rightarrow {}^4I_{15/2}$ emission pathway or Er^{3+} ions. We have observed that strong acid treatment of these PEI-UCNPs at least partially strips away the protective PEI and results in a loss of their luminescence, which we ascribe to upconversion luminescence quenching in water.[55]

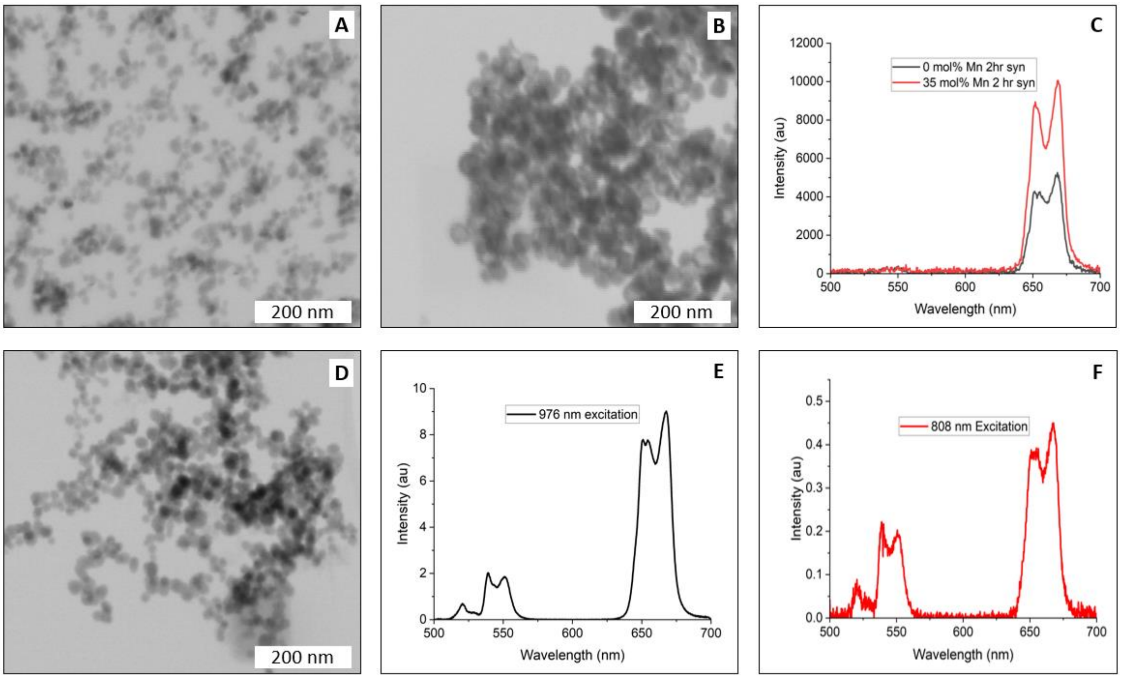

Figure S5. PEI-UCNPs produced as per described in Case Study #2. (A) STEM image of $NaYF_4$:Yb,Er PEI UCNPs (Yb = 18 mol %, Er = 2 mol %). (B) STEM images of $NaYF_4$:Yb,Er,Mn UCNPs (Yb = 18 mol %, Er = 2 mol %, Mn = 35 mol %) exhibiting potentially hollow structures. (C) Upconversion emission spectra of these UCNPs at 10 mg/mL in deionised water under 976 nm excitation.[ii] (D) STEM image of $NaYF_4$:Yb,Er@$NaYF_4$:Yb,Nd core/shell PEI UCNPs. (E) Emission of 1.5 mg/mL $NaYF_4$:Yb,Er@$NaYF_4$:Yb,Nd core/shell PEI UCNPs (core: Yb^{3+} 20 mol %, Er^{3+} 2 mol %; shell core: Yb^{3+} 20 mol %, Nd^{3+} 10 mol %) dispersed in water and excited at 976 nm. (F) Emission of same UCNPs as (E), excited at 808 nm. Thus demonstrating dual-band excitation in water.[iii]

[ii] These spectra were measured on a relatively low-cost modular upconversion spectrometer system consisting of a 900 mW 976 nm laser (BL976-PAG900 mounted on a CLD1015, Thorlabs), fibre optic delivery and collimating lens, a sample holder, short-pass filter (< 700 nm) fibre optic output, and a CCD spectrometer (OCEAN-HDXXR, Ocean Insight). This low-cost upconversion spectrometer has a poorer signal to noise ratio than well optimised upconversion spectrometers.

[iii] Measured with the upconversion spectroscopy system previously reported in Birch et al., (2023).[11]

SUPPLEMENTARY INFORMATION

In a small exploratory study, we introduced Mn^{2+} co-doping (Yb^{3+} = 18 mol %, Er^{3+} = 2 mol %, Mn^{2+} = 35 mol %) into PEI-UCNP synthesis with the aim of increasing red emission in a manner similar to our prior work with PVP-UCNPs (i.e. via constricting unit cell diameter and enabling a three-photon sensitization pathway).[12] As expected, we found that 35 mol% Mn^{2+} co-doping increased PEI-UCNP red band emission by approximately a factor of 3 over standard $NaYF_4$:Yb,Er PEI UCNPs (see Figure S5c). However, electron microscopy revealed that the Mn^{2+} co-doped PEI-UCNPs were not uniform in nature, rather they appeared to be hollow and larger than PEI-UCNPs without Mn^{2+} (see Figure S5a,b). These initial results may indicate that Mn^{2+} may bind to PEI in a way that alters resulting PEI-UCNP morphology; this may be consistent with the less-regular UCNPs observed when Mn^{2+} is used in co-doping in synthesis of PVP-UCNPs,[12] and hollow cubic-shaped OA-UCNPs.[59,60] Further study is required to investigate such effects.

Despite the advantage of immediate water dispersibility inherent to PEI-UCNPs, the number of papers published for PEI-UCNP synthesis seems to be considerably less than OA-UCNPs. A variety of dopant combinations have been explored for PEI-UCNPs to produce various emission colours, including Tm^{3+} to achieve higher-energy blue PEI-UCNP emission.[61–63,63] Some notable examples include use of Gd to achieve green emission,[64,65] achieving red and green emission by varying ratio of Yb^{3+} to Er^{3+},[66] and achieving both red and blue emission by varying ratio of Yb^{3+} to Tm^{3+}.[66] Intriguingly, Hu et al., (2014) [67] demonstrated that simply varying the amount of water in the solvothermal PEI-UCNP synthesis can select for production of cubic-phase PEI-$NaYF_4$;Yb,Er UCNPs (strong red emission) or hexagonal-phase PEI-$NaYF_4$;Yb,Er UCNPs (strong green emission) via reaction in a 25 mL PTFE-lined autoclave at 200 °C for 10 hours.[67] Some studies have formed silica shells around PEI-UCNPs, but to the best of our knowledge, mesoporous silica shells have not yet been formed around PEI-UCNPs.[65]

In another exploratory study, we adapted the PEI-UCNP methodology to enable dual-wavelength 808 nm and 976 nm excitation by attempting to an Nd^{3+} doped shell to the PEI-UCNPs. The resulting $NaYF_4$:Yb,Er@$NaYF_4$:Yb,Nd core/shell PEI UCNPs are shown in Figure S5d with corresponding emission spectra in Figure S5E and Figure S5F. This initial data is promising, but further well-resourced study is required to optimize these UCNPs, confirm shell formation, examine dopant distribution, and to fully characterize their photophysical properties. Nevertheless, PEI-UCNPs appear to be a rather straight-forward route to small-diameter water-dispersible UCNPs with multi-wavelength capabilities.

Whilst the reliability of PEI-UCNP synthesis is favorable, we have noticed one issue which results in failed synthesis: over-hydrated/wet hygroscopic staring reagents. Being situated in Scotland, our laboratory is a high-humidity environment, therefore various hygroscopic reagents used in the synthesis (i.e. rare earth nitrate hydrates, ammonium fluoride, and ethylene glycol) will absorb atmospheric moisture. This will change the carefully balanced stoichiometry of input materials over time, which manifests as a gradual reduction of UCNP emission intensity, eventually leading to optically-inert nanoparticle formation. Our solution to this is simply to periodically purchase fresh reagents.

In summary, PEI-UCNPs offer a reliable route to water-dispersible UCNPs that seem particularly favourable for biophotonic applications. For example, a number of studies have demonstrated that PEI-UCNPs have good biocompatibility and can be taken up by cells and tissues.[14,58,68] Jin et al., (2011) established that PEI-UCNPs are taken up via a clathrin endocytic mechanism.[14] Further, the highly positively charged PEI itself could be exploited to bind various molecules and proteins non-specifically (by simple electrostatic interactions) or specifically (by rationale-controlled organic modification of the PEI polymer). Overall, PEI-UCNP seems to be a highly reliable method of producing water dispersible UCNPs via solvothermal autoclave reactions and there is scope for future studies to push the boundaries of what is possible for PEI-UCNPs.

SUPPLEMENTARY INFORMATION

Supplementary Bibliography

1. Auzel, F. History of upconversion discovery and its evolution. *Journal of Luminescence* **223**, 116900 (2020).
2. Quintanilla, M. *et al.* Cubic versus Hexagonal – Phase, Size and Morphology Effects on the Photoluminescence Quantum Yield of NaGdF 4 :Er 3+ /Yb3+ Upconverting Nanoparticles. *Nanoscale* (2022) doi:10.1039/d1nr06319g.
3. Zhou, M. *et al.* The Bioavailability, Biodistribution, and Toxic Effects of Silica-Coated Upconversion Nanoparticles in vivo. *Frontiers in Chemistry* **7**, (2019).
4. Shao, B. *et al.* A novel synthetic route towards monodisperse β-NaYF4:Ln3+ micro/nanocrystals from layered rare-earth hydroxides at ultra low temperature. *Chemical Communications* **50**, 12706–12709 (2014).
5. Jiao, Y. *et al.* Controllable Synthesis of Upconversion Nanophosphors toward Scale-Up Productions. *Particle and Particle Systems Characterization* **37**, 1–13 (2020).
6. Lei, P. *et al.* Ultrafast Synthesis of Novel Hexagonal Phase NaBiF4 Upconversion Nanoparticles at Room Temperature. *Advanced Materials* **29**, 4–7 (2017).
7. Du, P., Luo, L., Huang, X. & Yu, J. S. Ultrafast synthesis of bifunctional Er 3+ /Yb 3+ -codoped NaBiF 4 upconverting nanoparticles for nanothermometer and optical heater. *Journal of Colloid and Interface Science* **514**, 172–181 (2018).
8. Liang, X., Wang, X., Zhuang, J., Peng, Q. & Li, Y. Synthesis of NaYF4 Nanocrystals with Predictable Phase and Shape. *Advanced Functional Materials* **17**, 2757–2765 (2007).
9. Lei, P. & Zhang, Y. Hollow upconversion nanoparticles: Synthesis and luminescence in comparison with their solid counterparts. *Chemical Engineering Journal* **426**, 131376 (2021).
10. Li, Z. & Zhang, Y. Monodisperse silica-coated polyvinyl-pyrrolidone/NaYF4 nanocrystals with multicolor upconversion fluorescence emission. *Angewandte Chemie - International Edition* **45**, 7732–7735 (2006).
11. Birch, R. *et al.* Influence of polyvinylpyrrolidone (PVP) in the synthesis of luminescent NaYF4:Yb,Er upconversion nanoparticles. *Methods Appl. Fluoresc.* **11**, 034001 (2023).

12. Mackenzie, L. E., Alvarez-ruiz, D. & Pal, R. Low-temperature open-air synthesis of PVP-coated NaYF 4 : Yb , Er , Mn upconversion nanoparticles with strong red emission. *Royal Society Open Science* **9**, 211508 (2022).
13. Zhu, X. *et al.* Large-molecular-weight PVP-functionalized stacked upconversion nanoplates for high-performance individual marking. *Cell Reports Physical Science* **4**, 101572 (2023).
14. Jin, J. *et al.* Polymer-Coated NaYF4:Yb3+, Er3+ Upconversion Nanoparticles for Charge-Dependent Cellular Imaging. *ACS Nano* **5**, 7838–7847 (2011).
15. Bower, J. S., Broughton, G. F. J., Stedman, J. R. & Williams, M. L. A winter NO2 smog episode in the U.K. *Atmospheric Environment* **28**, 461–475 (1994).
16. Stepuk, A., Casola, G., Schumacher, C. M., Krämer, K. W. & Stark, W. J. Purification of NaYF4-Based Upconversion Phosphors. *Chem. Mater.* **26**, 2015–2020 (2014).
17. Li, C., Yang, J., Yang, P., Lian, H. & Lin, J. Hydrothermal Synthesis of Lanthanide Fluorides LnF3 (Ln = La to Lu) Nano-/Microcrystals with Multiform Structures and Morphologies. *Chem. Mater.* **20**, 4317–4326 (2008).
18. Wang, H. Q. & Nann, T. Monodisperse upconverting nanocrystals by Microwave-assisted synthesis. *ACS Nano* **3**, 3804–3808 (2009).
19. *MARS 6 Microwave Reaction System Operation Manual*. (2011).
20. Amouroux, B. *et al.* Importance of the Mixing and High-Temperature Heating Steps in the Controlled Thermal Coprecipitation Synthesis of Sub-5-nm Na(Gd-Yb)F 4 :Tm. *Inorganic Chemistry* **58**, 5082–5088 (2019).
21. Egatz-Gomez, A., Asher, M., Peterson, R., A. Roldan, M. & Ros, A. Microwave synthesis of upconverting nanoparticles with bis(2-ethylhexyl) adipate. *RSC Advances* **12**, 23026–23038 (2022).
22. Panov, N., Marin, R. & Hemmer, E. Microwave-Assisted Solvothermal Synthesis of Upconverting and Downshifting Rare-Earth-Doped LiYF4 Microparticles. *Inorg. Chem.* **57**, 14920–14929 (2018).
23. Reddy, K. L. *et al.* Amine-functionalized, porous silica-coated NaYF4:Yb/Er upconversion nanophosphors for efficient delivery of doxorubicin and curcumin. *Materials Science and Engineering: C* **96**, 86–95 (2019).

24. Halimi, I. *et al.* Pick your precursor! Tailoring the size and crystal phase of microwave-synthesized sub-10 nm upconverting nanoparticles. *Journal of Materials Chemistry C* **7**, 15364–15374 (2019).
25. Homann, C. *et al.* Gum Arabic-stabilized upconverting nanoparticles for printing applications. *Optical Materials: X* **21**, 100290 (2024).
26. Nair, G. B., Tamboli, S., Kroon, R. E. & Swart, H. C. Microwave-assisted hydrothermal synthesis of LaOF:Yb3+, Ho3+ nanorods with high thermoresponsive upconversion luminescence for thermometry. *Materials Today Chemistry* **29**, 101463 (2023).
27. Wang, H. & Nann, T. Monodisperse upconversion GdF3:Yb, Er rhombi by microwave-assisted synthesis. *Nanoscale Res Lett* **6**, 267 (2011).
28. Thodhal Yoganandham, S. *et al.* Mineral and Trace Metal Concentrations in Seaweeds by Microwave-Assisted Digestion Method Followed by Quadrupole Inductively Coupled Plasma Mass Spectrometry. *Biol Trace Elem Res* **187**, 579–585 (2019).
29. Karasakal, A. Determination of Major, Minor, and Toxic Elements in Tropical Fruits by ICP-OES After Different Microwave Acid Digestion Methods. *Food Anal. Methods* **14**, 344–360 (2021).
30. Mun, K. R. *et al.* Elemental-Migration-Assisted Full-Color-Tunable Upconversion Nanoparticles for Video-Rate Three-Dimensional Volumetric Displays. *Nano Lett.* (2023) doi:10.1021/acs.nanolett.3c00397.
31. Cheng, T., Marin, R., Skripka, A. & Vetrone, F. Small and Bright Lithium-Based Upconverting Nanoparticles. *J. Am. Chem. Soc.* **140**, 12890–12899 (2018).
32. Homann, C. *et al.* NaYF4:Yb,Er/NaYF4 Core/Shell Nanocrystals with High Upconversion Luminescence Quantum Yield. *Angewandte Chemie International Edition* **57**, 8765–8769 (2018).
33. Zhou, Z. *et al.* Optical gain based on NaYF4: Er3+, Yb3+ nanoparticles-doped polymer waveguide under convenient LED pumping. *Applied Physics Letters* **118**, 173301 (2021).
34. Conibeer, G. Third-Generation Solar Cells. in *Solar Cell Materials* 283–314 (John Wiley & Sons, Ltd, 2014). doi:10.1002/9781118695784.ch9.
35. Skripka, A. *et al.* Decoupling Theranostics with Rare Earth Doped Nanoparticles. *Advanced Functional Materials* **29**, 1807105 (2019).

36. Sarkar, D., Meesaragandla, B., Samanta, T. & Mahalingam, V. A Greener Approach towards Making Highly Luminescent Ln3+-Doped NaYF4 Nanoparticles with Ligand-Assisted Phase Control. *ChemistrySelect* **1**, 4785–4793 (2016).
37. Andresen, E. *et al.* Assessing the reproducibility and up-scaling of the synthesis of Er,Yb-doped NaYF4-based upconverting nanoparticles and control of size, morphology, and optical properties. *Sci Rep* **13**, 2288 (2023).
38. You, W. *et al.* Large-scale synthesis of uniform lanthanide-doped NaREF4 upconversion/downshifting nanoprobes for bioapplications. *Nanoscale* **10**, 11477–11484 (2018).
39. Chan, E. M. *et al.* Combinatorial discovery of lanthanide-doped nanocrystals with spectrally pure upconverted emission. *Nano Letters* **12**, 3839–3845 (2012).
40. Zhao, Z. *et al.* Multifunctional Core–Shell Upconverting Nanoparticles for Imaging and Photodynamic Therapy of Liver Cancer Cells. *Chemistry – An Asian Journal* **7**, 830–837 (2012).
41. Ostrowski, A. D. *et al.* Controlled Synthesis and Single-Particle Imaging of Bright, Sub-10 nm Lanthanide-Doped Upconverting Nanocrystals. *ACS Nano* **6**, 2686–2692 (2012).
42. Chan, E. M., Gargas, D. J., Schuck, P. J. & Milliron, D. J. Concentrating and Recycling Energy in Lanthanide Codopants for Efficient and Spectrally Pure Emission: The Case of NaYF4:Er3+/Tm3+ Upconverting Nanocrystals. *J. Phys. Chem. B* **116**, 10561–10570 (2012).
43. Materia, M. E. *et al.* Multifunctional Magnetic and Upconverting Nanobeads as Dual Modal Imaging Tools. *Bioconjugate Chem.* **28**, 2707–2714 (2017).
44. Berkley Lab: Molecular Foundry. Solution Synthesis Robots. https://foundry.lbl.gov/instrumentation/solution-synthesis-robots/ (2023).
45. Xia, X., Sivonxay, E., Helms, B. A., Blau, S. M. & Chan, E. M. Accelerating the Design of Multishell Upconverting Nanoparticles through Bayesian Optimization. *Nano Lett.* **23**, 11129–11136 (2023).
46. Bateni, F. *et al.* Smart Dope: A Self-Driving Fluidic Lab for Accelerated Development of Doped Perovskite Quantum Dots. *Advanced Energy Materials* **14**, 2302303 (2024).

47. Bi, S., Deng, Z., Huang, J., Wen, X. & Zeng, S. NIR-II Responsive Upconversion Nanoprobe with Simultaneously Enhanced Single-Band Red Luminescence and Phase/Size Control for Bioimaging and Photodynamic Therapy. *Advanced Materials* **35**, 2207038 (2022).
48. Chia, S. W. & Misran, M. Flow Behavior of Oleic Acid Liposomes in Sucrose Ester Glycolipid Oil-in-Water Emulsions. *J Surfact Deterg* **17**, 1–10 (2014).
49. Zhang, F. *et al.* Uniform Nanostructured Arrays of Sodium Rare-Earth Fluorides for Highly Efficient Multicolor Upconversion Luminescence. *Angewandte Chemie* **119**, 8122–8125 (2007).
50. Mai, H.-X., Zhang, Y.-W., Sun, L.-D. & Yan, C.-H. Size- and Phase-Controlled Synthesis of Monodisperse NaYF4:Yb,Er Nanocrystals from a Unique Delayed Nucleation Pathway Monitored with Upconversion Spectroscopy. *J. Phys. Chem. C* **111**, 13730–13739 (2007).
51. Liu, C. & Chen, D. Controlled synthesis of hexagon shaped lanthanide-doped LaF3 nanoplates with multicolor upconversion fluorescence. *J. Mater. Chem.* **17**, 3875–3880 (2007).
52. Sui, Y., Tao, K., Tian, Q. & Sun, K. Interaction Between Y3+ and Oleate Ions for the Cubic-to-Hexagonal Phase Transformation of NaYF4 Nanocrystals. *J. Phys. Chem. C* **116**, 1732–1739 (2012).
53. Ding, M., Lu, C., Cao, L., Ni, Y. & Xu, Z. Controllable synthesis, formation mechanism and upconversion luminescence of β-NaYF4:Yb3+/Er3+ microcrystals by hydrothermal process. *CrystEngComm* **15**, 8366–8373 (2013).
54. Li, C. *et al.* Different Microstructures of β-NaYF4 Fabricated by Hydrothermal Process: Effects of pH Values and Fluoride Sources. *Chem. Mater.* **19**, 4933–4942 (2007).
55. Arppe, R. *et al.* Quenching of the upconversion luminescence of NaYF 4 :Yb 3+ , Er 3+ and NaYF 4 :Yb 3+ , Tm 3+ nanophosphors by water: the role of the sensitizer Yb 3+ in non-radiative relaxation. *Nanoscale* **7**, 11746–11757 (2015).
56. Mai, H.-X. *et al.* High-Quality Sodium Rare-Earth Fluoride Nanocrystals: Controlled Synthesis and Optical Properties. *J. Am. Chem. Soc.* **128**, 6426–6436 (2006).

57. Zhang, S. *et al.* Fluorescence resonance energy transfer between NaYF4:Yb,Tm upconversion nanoparticles and gold nanorods: Near-infrared responsive biosensor for streptavidin. *Journal of Luminescence* **147**, 278–283 (2014).
58. Nampi, P. P. *et al.* Selective cellular imaging with lanthanide based upconversion nanoparticles. *Journal of Biophotonics* e201800256 (2018) doi:10.1002/jbio.201800256.
59. Liu, Y. *et al.* Single band red emission of Er3+ ions heavily doped upconversion nanoparticles realized by active-core/active-shell structure. *Ceramics International* (2021) doi:10.1016/j.ceramint.2021.03.220.
60. Liu, Y. *et al.* Highly efficient upconversion single red emission of hollow cubic α-NaErF4 nanoparticles by Mn/Yb heavy doping. *Journal of Luminescence* **228**, 117637 (2020).
61. Xu, S. *et al.* A novel upconversion, fluorescence resonance energy transfer biosensor (FRET) for sensitive detection of lead ions in human serum. *Nanoscale* **6**, 12573–12579 (2014).
62. Zhang, D. *et al.* Mitochondrial specific photodynamic therapy by rare-earth nanoparticles mediated near-infrared graphene quantum dots. *Biomaterials* **153**, 14–26 (2018).
63. Liu, J. *et al.* Up-conversion fluorescence biosensor for sensitive detection of CA-125 tumor markers. *Journal of Rare Earths* **37**, 943–948 (2019).
64. Liu, R. *et al.* Development of a fluorescence aptasensor for rapid and sensitive detection of Listeria monocytogenes in food. *Food Control* **122**, 107808 (2021).
65. Ding, C. *et al.* Ratiometric Upconversion Luminescence Nanoprobe with Near-Infrared Ag2S Nanodots as the Energy Acceptor for Sensing and Imaging of pH in Vivo. *Anal. Chem.* **91**, 7181–7188 (2019).
66. Li, X. *et al.* New insight into modulated up-conversion luminescent silica nanotubes as efficient adsorbents for colored effluents. *Dalton Transactions* **43**, 15457–15464 (2014).
67. Hu, Y. *et al.* A facile synthesis of NaYF 4 :Yb 3+ /Er 3+ nanoparticles with tunable multicolor upconversion luminescence properties for cell imaging. *RSC Advances* **4**, 43653–43660 (2014).
68. Zhong, L. *et al.* Highly Retentive, Anti-Interference, and Covert Individual Marking Taggant with Exceptional Skin Penetration. *Advanced Science* **9**, 2201497 (2022).
